# Enhanced Treatment Effects of Tilmicosin Against *Staphylococcus aureus* Cow Mastitis by Self-Assembly Sodium Alginate-Chitosan Nanogel

**DOI:** 10.3390/pharmaceutics11100524

**Published:** 2019-10-12

**Authors:** Kaixiang Zhou, Xiaofang Wang, Dongmei Chen, Yuanyuan Yuan, Shuge Wang, Chao Li, Yuanyuan Yan, Qianying Liu, Liwei Shao, Lingli Huang, Zonghui Yuan, Shuyu Xie

**Affiliations:** 1 National Reference Laboratory of Veterinary Drug Residues (HZAU) and MAO Key Laboratory for Detection of Veterinary Drug Residues, Wuhan 430070, Hubei, China; flyingkai@webmail.hzau.edu.cn (K.Z.); yuanyuanyuan890@webmail.hzau.edu.cn (Y.Y.); shugewang@webmail.hzau.edu.cn (S.W.); chaoli@webmail.hzau.edu.cn (C.L.); yuanyuanyan@webmail.hzau.edu.cn (Y.Y.); liuqianying@webmail.hzau.edu.cn (Q.L.); 2 MOA Laboratory for Risk Assessment of Quality and Safety of Livestock and Poultry Products, Huazhong Agricultural University, Wuhan 430070, Hubei, China; chendongmei@mail.hzau.edu.cn (D.C.); huanglingli@mail.hzau.edu.cn (L.H.); yuan5802@mail.hzau.edu.cn (Z.Y.); 3 Animal husbandry and veterinary institute of Hebei Province, Dongguan Street 428, Baoding 071000, Hebei, China; wangxiao.fang99@163.com (X.W.); slw15176206238@163.com (L.S.)

**Keywords:** tilmicosin, solid lipid nanoparticles, sodium alginate-chitosan nanogel, *Staphylococcus aureus*, cow mastitis

## Abstract

The *Staphylococcus aureus* (*S. aureus*) cow mastitis causes great losses to the cow industry. In order to improve the treatment effect of tilmicosin against cow mastitis, the combination of solid lipid nanoparticle (SLN) technology with in situ hydrogel technology was used to prepare the self-assembly tilmicosin nanogel (TIL-nanogel). The physicochemical characteristics, in vitro release, antibacterial activity and in vivo treatment efficacy of TIL-SLNs and TIL-nanogel were studied, respectively. The results showed the loading capacity (LC), encapsulation efficiency (EE), size, zeta potential and poly dispersion index (PDI) of TIL-nanogel were 23.33 ± 0.77%, 67.89 ± 3.01%, 431.57 ± 12.87 nm, 8.3 ± 0.06 mv and, 0.424 ± 0.032, respectively. The TIL-nanogel showed stronger sustained release in vitro than TIL-SLNs and commercial injection. The cure rate of half dosage and normal dosage of TIL-nanogel was 58.3% and 75.0%, which was higher than that of commercial injection (50.0%) at normal dosage. The results suggest that the treatment dosage of tilmicosin for cow mastitis could be reduced by TIL-nanogel. The novel TIL-nanogel will be beneficial by decreasing the usage of tilmicosin and the treatment costs of cow mastitis.

## 1. Introduction

Bovine mastitis is a microbiology infectious inflammation of the mammary gland and one of the key and serious harmful pathogens is *Staphylococcus aureus* (*S. aureus*). It was reported that the proportion of *Staphylococcal* subclinical mastitis is 30% of cow mastitis [1]. The cow mastitis induced by *S. aureus* has led to several economic losses in cow industry, for example, the reduction of milk yield and quality, and an increase the slaughter rate and mortality of cows [2,3]. It was reported that the cost of mastitis treatment was about $200 per cow per year [4]. Because of the *S. aureus* infections, the loss of milk was nearly 380 tons per year in the world [5]. Meanwhile, the presence of *S. aureus* in raw milk is also an important public health problem through the food chain. Currently, the situation of *S. aureus* bovine mastitis is severe and without available effective methods for its therapy.

Tilmicosin is a kind of semi-synthetic macrolide antibiotic with antibacterial activity against *Mycoplasma* spp., *Pasteurella* spp. and a variety of gram-positive bacteria [6]. Because it concentrates in the udder efficiently, tilmicosin is suggested to be applied to the treatment of mastitis in dairy cows [7]. Ziv et al. reported that the minimum inhibitory concentration (MIC_90_) of tilmicosin against 112 *S. aureus* isolated from the cow mastitis was 0.78 μg/mL [8]. The clinical therapy study indicated that the cure rate of *S. aureus* cow mastitis by mammary perfusion of tilmicosin could reach above 90% [9]. It was proved that administrating tilmicosin to cows at a dosage of 20 mg/kg by subcutaneous was useful for the treatment of intramammary infections caused by *S. aureus* [10]. Meanwhile, Martínez-Cortés et al. proved that tilmicosin could be used as an effective modulator of inflammation in the mammary gland, since tilmicosin could prevent cells from being damaged and help to maintain normal physiological functions of the cow mammary epithelial cell [11]. According to reports, the typical characteristics of *S. aureus* cow mastitis are chronic infection and reinfection [12,13]. Therefore, the therapy preparations of cow mastitis are preferred to have a long-term effect. Tilmicosin has long post-antibiotic effects [14]. The time of local drug concentrations above minimum inhibitory concentration (MIC) (T > MIC) is important to obtain a satisfactory clinical efficacy for tilmicosin [15] thus the sustained release preparation of this drug was preferred. 

However, *S. aureus* is a kind of common facultative intracellular bacteria, when *S. aureus* enters into cells, the cell membrane will protect it from detection by the immune system and being attacked by antimicrobial agents and then the bacterial reservoirs which link with reinfections and chronic infection will also be established [13,16]. The intracellular survival nature of *S. aureus* is one of the key reasons why cow mastitis caused by *S. aureus* is so hard to cure. One of the necessary conditions to treat intracellular infections successfully is to keep a satisfactory concentration of antimicrobial agents at the intracellular infected site [17]. However, tilmicosin shows short intracellular retention time [18], which will lead to low intracellular drug concentration and then failed treatment of cow mastitis will occur. Faced with the challenges of the intracellular survival of *S. aureus* and the short intracellular retention time of tilmicosin, development of a new kind of drug delivery system of tilmicosin with sustained release, long intracellular retention time and various antibacterial mechanisms is necessary.

Solid lipid nanoparticles (SLNs) are a kind of novel core-shell structure nanosized drug delivery system which make use of high-melting natural or synthetic solid lipids (e.g., long-chain fatty acids, wax, triglyceride) as backbone materials [19]. Common preparation methods are hot melt emulsification or solvent evaporation method. Because of the high permeability and small size of SLNs, they can easily penetrate cells and bacterial biofilms compared to free drugs [19]. After entering host cells, because of the good biocompatibility of lipid carriers, they can remain in the subcellular structure and improve the intracellular concentration of their payload antimicrobial agents. Our previous work indicated that the amount of accumulation and storage time of enrofloxacin within cells was effectively improved by enrofloxacin-loaded docosanoic acid SLNs [20]. Kalhapure et al. proved that vancomycin HCL (hydrochloric)-SLNs showed higher and longer efficacy to the resistant and sensitive *S. aureus* than vancomycin HCL [21]. Meanwhile, our previous work demonstrated that tilmicosin-loaded hydrogenated castor oil (HSO) SLNs enhanced the therapeutic efficacy of tilmicosin against a mouse mastitis model by determination with lower CFU (colony-forming units) counts [6]. However, because of the low uptake ability of mammary epithelial cells (non-professional phagocytes), they will limit the transmembrane transport of tilmicosin-loaded SLNs (TIL-SLNs). Therefore, the ability of single TIL-SLN penetrating mammary epithelial cells may not receive satisfactory results. 

Hydrogels refer to a solution, suspension, emulsion type of viscous liquid or semi-solid preparation made from drugs and polymers which can form a gel (e.g., chitosan, sodium alginate, carbopol, gelatin and others). It so happens that the polymer can improve the uptake ability of cells. Zhang et al. indicated that insulin trimethyl chitosan (degree of deacetylation: >90%, MW (molecular weight): 400 kDa) nanoparticles could improve the insulin amount into the Caco-2 cell via internalization compared to an insulin solution [22]. There was some research which showed that some polymers have some positive medical function, e.g., use as the sustained drug carrier material and promotion materials of wound healing [23], especially, sodium alginate (SA, 105 kDa, G/M (GulA/ManA) ratio = 0.85) and chitosan (MW: 134 kDa, degree of deacetylation: 96.44%) which have been used as advanced wound dressings in the clinic [24]. Also, some gel materials have an antibacterial activity such as chitosan [25,26]. The -NH_2_ group of chitosan makes the nanogel show positive charge in the acid which can lead to negative bacterial cell membrane lysis via charge interaction [27]. An in vitro checker board assay showed that the 2.6 kDa chitosan produced a synergy with the macrolides (e.g., tilmicosin) and reduced the MIC of both molecules by 2–8 times [28]. However, for chitosan antimicrobial activity to work it requires a higher concentration. In order to obtain a higher chitosan concentration in a cow’s mammary, the carboxymethyl chitosan with large solubility was applied in our research. Nanogels are nanosized hydrogels [29]. Because a polymer can enhance cellular uptake, promote wound healing and have antibacterial function, nanogels have the advantages of both nanoparticles and hydrogel. The vancomycin chitosan folic acid nanoparticles showed the more effective performance across epithelial and bacterial cell surfaces and stronger anti-*S. aureus* effect compared with vancomycin nanoparticles [30].

**Figure 1:**
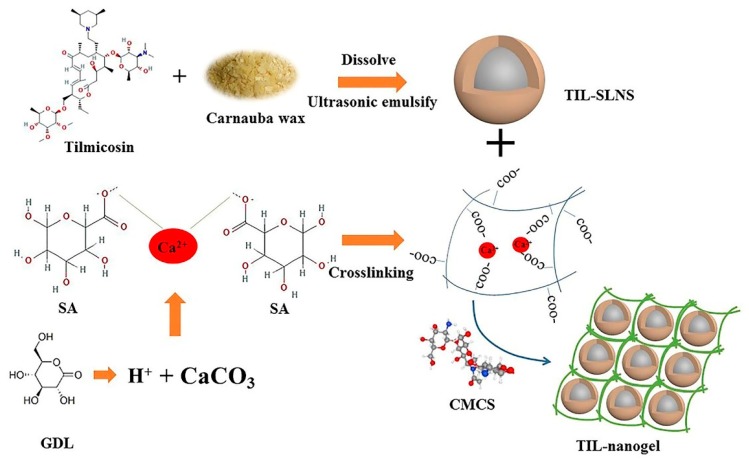
The preparation process of tilmicosin-loaded solid lipid nanoparticles (TIL-SLNs) and TIL-nanogel. CMCS, carboxymethyl chitosan; GDL, gluconolactone; SA, sodium alginate.

According to the report of Sivaram et al. there are two measures to prepare nanogel: (a) direct chemical or physical crosslinking; (b) preparation of the nanogel based on nanoparticle suspension (e.g., liposomes, nanoemulsion) [29]. Taking the residues of organic reagents into consideration in chemical crosslinking, the physical crosslinking method without organic reagents was preferred to prepare hydrogel. The sequence of SA crosslinking with multivalent cations is Pb^2+^ > Cu^2+^ > Cd^2+^ > Ba^2+^ > Sr^2+^ > Ca^2+^ > Co^2+^ [31]. Although the crosslinking ability of Pb^2+^ and Cu^2+^ is higher than Ca^2+^, the biocompatibility of Ca^2+^ is higher. Compared with using CaCl_2_ as the crosslinking agent, the in situ gelling method, a self-assembly period was preferred, due to the lower mechanical force requirement. Briefly, the glucuronide (GDL) was used as the H^+^ source. The H^+^ was slowly released from the GDL solution to react with CaCO_3_ to produce Ca^2+^. Then the sustained release Ca^2+^ would crosslink with SA spontaneously to form uniform hydrogel rather than under mechanical stirring or ultrasonication [32] (Figure 1). If the formed hydrogel particles are added into the chitosan solution, the chitosan coated SA nanogel or microparticles can be obtained by the reaction between negative charge of SA and positive charge of chitosan [33,34]. As mentioned above, the SA-chitosan nanogel could have the double advantages of being antibacterial and promoting wound healing.

In view of these, a kind of novel tilmicosin nanogel (TIL-nanogel) was prepared by combination of SLN technology with self-assemble hydrogel technology in our paper. The characteristics, stability, sustained release performance in vitro, in vitro antibacterial activity and the clinical treatment effects of TIL-nanogel were further studied.

## 2. Methods and Animals

### 2.1. Materials

Tilmicosin (content: >96%) was obtained from Jinan Xinbao Star Animal Pharmaceutical Co., Ltd. (Jinan, China). Tilmicosin standard (content: 80.7%) was purchased from Dr. Ehrenstorfer (Shanghai, China). Tilmicosin injection (10 mL:3 g) was provided by ChuangXin Pharmaceutical Co., Ltd. (Jiangxi, China). Carnauba wax (melt point: 83 °C) was provided by Aladdin (Shanghai, China). Carboxymethyl chitosan (CMCS, CAS (chemical abstracts service): 83512-85-0, carboxylation degree: 82.6%, solubility: ≥50 mg/mL, MW: 194.6 kDa g/moL, degree of carboxyl substitution: 108.41%, degree of deacetylation: 90%) and poly vinyl alcohol (PVA) were obtained from Source Biological Technology Co., Ltd. (Shanghai, China). Sodium alginate (SA, CAS: 9005-38-3, MW: 150 kDa, M/G ratio: 0.89), Na_2_HPO_4_, KH_2_PO_4_, KCl, NaCl, Calcium carbonate (CaCO_3_) and citric acid were purchased from Sinopharm Group Chemical Reagent Co., Ltd. (Shanghai, China). Gluconolactone (GDL) was bought from Angel Yeast Co., Ltd. (Yichang, China). Mannitol salt agar was provided by Hopebio Co., Ltd. (Qingdao, China).

### 2.2. Animals

Seventy-two China Holstein cows (400–500 kg) with somatic cell counts (SCC) higher than 500,000 cells/mL were kept at Haoyu Animal Husbandry Co., Ltd. (Baoding, China). The cows were fed drug-free feed and were drinking freely. All the experimental animal protocols were authorized by the Institutional Animal Care and Use Committee at Huazhong Agricultural University (Approval number HZAUSW-2018-041, 5 April 2019) and followed the guidelines of Hebei Science and Technology.

### 2.3. Bacteria

The *S. aureus* clinical strains (No.1687) were collected from the clinic and stored in the National Reference Laboratory of Veterinary Drug Residues (HZAU). 

### 2.4. Preparation of TIL-SLNs and TIL-Nanogel

The preparation processes of TIL-SLNs and TIL-nanogel were as shown in Figure 1. Firstly, TIL-SLNs were prepared by a hot homogenization and ultrasonication method which were introduced in our previous paper [35]. Briefly, 2 g carnauba wax and 1 g tilmicosin were added in a 25 mL tube and heated at 95 °C. After that the lipid was melted and the drug was dissolved into the carnauba wax, 25 mL 3% PVA water solution which contained 150 mg SA and 150 mg GDL, pre-heated in a boiling water bath, was poured into the lipid phase under magnetic stirring, then sonicated (VCX 130 Vibra-Cell^TM^, Sonics & Materials, Inc., Newtown, CT, USA) to obtain the TIL-SLNs suspension. When the TIL-SLNs suspension was cooled down to room temperature, 250 mg CaCO_3_ was added. It was kept static for 1 h after stirring for 10 min to make sure that the added CaCO_3_ completely reacted with the H^+^ which was provided by the GDL. Then, the slow-released Ca^2+^ would crosslink with SA spontaneously. Subsequently, 125 mg CMCS was added under stirring for 10 min to mix them thoroughly and then kept static overnight at 25 °C to let the CMCS react with the SA completely to obtain the TIL-nanogel.

### 2.5. Characteristics of TIL-SLNs and TIL-Nanogel

#### 2.5.1. Loading Capacity and Encapsulation Efficiency

The method of determining loading capacity (LC) and encapsulation efficiency (EE) was introduced in the previous literature [20,35]. Briefly, 10 mg freeze-dried nanogel encapsulated tilmicosin was added to a 10 mL tube containing 8 mL acetonitrile/water solution (*v*/*v*; 1:1) and put in a boiling water bath for 20 min to destroy the nanogel and release the inner tilmicosin. After boiling, the volume was added to an 8 mL tube and centrifuged at 12,000 rpm (Hitachi Centrifuge CR21G; Hitachi Koki Co., Ltd., Tokyo, Japan) for 15 min. The supernatant was diluted and injected into Waters 2695 series HPLC equipped with a UV detector (Waters Corp., Milford, MA, USA) for analysis after filtration. The assay was repeated three times using different samples from independent preparations. The loading capacity (LC) and encapsulation efficiency (EE) were defined as follows:

LC (%) = [(Weight of tilmicosin in SLNs)/(Weight of SLNs)] × 100%
EE (%) = [(Weight of tilmicosin in SLNs)/(Weight of tilmicosin added)] × 100%

#### 2.5.2. Quantitative Measurement of Tilmicosin

Tilmicosin concentration was measured using HPLC (Waters group, USA). The chromatographic conditions were: Column: (C_18_, 250 mm × 4.6 mm × 5 μm); detection wavelength: 287 nm; column temperature: 25 °C; mobile phase: water (phase A), acetonitrile (phase B), methanol (phase C) and 0.2 M ammonium formate (phase D, pH = 5.0) with the ratio of 32:24:24:20; flow rate: 1.00 mL/min; and injection volume: 10 μL. The content of tilmicosin was determined according to a standard curve. The HPLC method was validated in terms of linearity, accuracy, precision and limitation of detection. The linear range was from 1 to 10 μg/mL (*R*^2^ = 0.9996). The relative SD of precision was <2%, and the recovery rates were 95.6–102.4%.

#### 2.5.3. Analysis of Size, Poly Dispersion Index (PDI) and Zeta Potential

The size, PDI and zeta potential of TIL-SLNs and nanogel were measured by photon correlation spectroscopy (PCS) by using Zetasizer ZX3600 (Malvern Instruments, Worcestershire, UK) at 25 °C. All measurements were repeated in triplicate by using different samples from independent preparations. 

#### 2.5.4. Scanning Electron Microscope (SEM) and Differential Scanning Calorimeters (DSC) Analysis

For SEM analysis, 10 μL samples were suspended in 5 mL distilled water and 2 μL of the suspension was placed on a cover glass. The samples after oven-drying were fixed on an SEM stub and coated with gold at 20 mA for 2 min using an autofine coater (Ion sputter JFC 1600, JEOL, Tokyo, Japan). After coating, the samples were observed by the SEM with a secondary electron detector with an accelerating voltage of 15 kV.

The thermal analysis of pure tilmicosin, TIL-SLNs and TIL-nanogel were performed by the DSC (DSC200PC, NETZSCH, Selb, Germany). Briefly, under the nitrogen purge, 3 mg samples were heated from 25 to 250 °C with a heating rate of 10 °C/min. 

#### 2.5.5. In Vitro Release Studies

The in vitro release was performed at pH = 7.5 phosphate buffer solution (PBS). Briefly, 0.6 mL tilmicosin injection, 3 mL TIL-SLNs and 3 mL TIL-nanogel (containing 60 mg tilmicosin) were added to a dialysis bag (molecular weight: 20,000) and dialyzed against 500 mL PBS solution (receiver solution) in a 500 mL dissolution cup at 38 ± 0.5 °C using a dissolution tester stirring at 60 rpm to imitate the surroundings of a cow mammary gland. To determine the amount of tilmicosin diffused through the dialysis bag, a 1 mL sample was withdrawn from the receiver solution and 1 mL of fresh medium was added to maintain a constant volume. The fresh PBS was used to replace the receiver solution every 24 h to let the tilmicosin release completely. The experiments were carried out in triplicate and the samples were measured by HPLC. Then the cumulative release curve was drawn by Graphpad prism (version 7.0). The cumulative release of tilmicosin was determined by the following equation [36]:

Cumulative release of tilmicosin (%) = *R*_t_/*L* × 100

where *L* and *R_t_* represent the initial amount of tilmicosin loaded and cumulative amount of tilmicosin released at time *t*, respectively.

#### 2.5.6. Stability Evaluation

The stability of TIL-SLNs and TIL-nanogel was evaluated by an influencing factor experiment including high temperature, high humidity and strong light (ICH Topic Q1A (R2)). Briefly, the TIL-SLNs and TIL-nanogel were placed in a container and then placed in an environment of 40 °C (high temperature test), 25 °C and 90% ± 5% (humidity test) or 4500 ± 500 lx (high light test) for 10 days. Samples were taken on the 5th and 10th day to assess the change of the size, LC, appearance and drug content.

### 2.6. Antibacterial Activity

The in vitro antibacterial activity of tilmicosin preparations was determined by the agar diffusion method. Briefly, 15 mL agar medium was first poured into an aseptic plate, then once the 15 mL medium agar had solidified, another 5 mL agar medium which contained 0.1 mL bacterial fluid (contained 10^5^–10^6^ CFU *S. aureus*) was poured on the 15 mL agar medium. When the agar was solid, a suitable straw was used to punch four holes in the solid agar. Then 50 μL physiological saline, tilmicosin standard, TIL-SLNs and TIL-nanogel (tilmicosin content: 8 μg/mL) were added. Physiological saline was used as the control. The plates incubated *S. aureus* were put in an incubator (5% CO_2_, 37 °C). After culturing for 24 h, the size of the inhibition zones was measured and recorded.

### 2.7. Evaluation of Clinical Cow Mastitis Treatment Effects

The cows with *Staphylococcal* mastitis were diagnosed via the California mastitis test (CMT), detection of SCC by the Dairy Herd Improvement (DHI) center of Hebei province and by *S. aureus* isolation from milk samples. If the SCC > 500,000 cells/mL and the *S. aureus* was recognized, the cows could be selected into the treatment experiment. Seventy-two cows with naturally infected *S. aureus* were randomly divided into six groups including the control group, commercial tilmicosin injection group, low TIL-SLNs group, normal TIL-SLNs group, low TIL-nanogel group and normal TIL-nanogel group. The treatment was administered continuously for 5 days with a dosage of 150 or 300 mg tilmicosin/mammary per day (Table 1). Before administration, the mammary gland was disinfected by 0.15% povidone-iodine and 75% ethanol then warmed by a hot towel (53 °C). After administration, the mammary glands were massaged to let the drug diffuse completely. The milk samples were collected the day before administration, the 6th day and the 13th day to analyze their pH, number of *S. aureus* and SCC, respectively. Meanwhile, the milk yield (milking twice a day) at the above time points was recorded to analyze the milk yield of each cow of every group after treatment. Finally, according to the clinical symptoms and the pH, *S. aureus* count, SCC values of milk samples and the milk yield to comprehensively evaluate the therapeutic effects of various groups. According to the reports and the requirement of Ministry of Agriculture of China, among these factors, the SCC and *S. aureus* counts were the main indexes to evaluate whether the treatment methods were effective (*S. aureus* was not recovered from previously infected quarters and SCC < 500,000 cells/mL), invalid (SCC > 500,000 cells/mL) or the *S. aureus* mastitis were cured (*S. aureus* was not recovered from previously infected quarters and SCC < 200,000 cells/mL) [37,38]. 

### 2.8. Statistical Analysis

Data are presented as mean ± standard deviation (SD). The one-way analysis of variance and chi-square test were adopted for statistical analysis. Statistical significance was defined at a *p*-value of 0.05 by the SPSS software (version 20, IBM, New York, NY, USA).

## 3. Results and Discussion

### 3.1. The Formulation of TIL-Nanogel

In order to obtain the TIL-nanogel with higher content, the carnauba wax which has higher solubility for tilmicosin (50%, 100 °C) was selected as the lipid material rather than HCO (25%, 100 °C), as reported in our previous study [6]. Then the formulation of TIL-SLNs was optimized by orthogonal design (Supplementary Tables S1 and S2). After the TIL-SLNs suspension was obtained, the influence factor tests were performed to determine the stability of TIL-SLNs. The result showed that the TIL-SLNs was not stable in the basicity (Table 2). Therefore, the GDL was added as an acidity regulator. To keep the weak acidity for the complete reaction of CaCO_3_, 150 mg GDL was added. To obtain the nanosized gel, 150 mg SA and 125 mg CMCS was added. As mentioned above, nanogel could be obtained by direct chemical or physical crosslinking or produced on the basis of nanoparticle suspension [33]. In view of the requirement for less mechanical force, the TIL-nanogel was prepared by the latter method in our paper. In contrast to drop CaCl_2_ solution as the crosslinking agent, the in situ gelling method, a self-assembly period was adopted. Briefly, the H^+^ was slowly released from the GDL solution to react with CaCO_3_ to produce Ca^2+^. Then the sustained release Ca^2+^ would crosslink with SA to form a uniform hydrogel [32] (Figure 1). After CMCS was added, the CMCS coated SA nanogel would be obtained by the interaction of the positive charge of CMCS and the negative charge of SA. This self-assembly process was a spontaneous process in which less mechanical force was needed (such as stirring or ultrasonication) and thus could prevent the nanoparticles from being destroyed. Because the self-assembly method could protect the drugs, in the field of delivery of macromolecular drugs especially for genes and proteins, the self-assembly technologies of hydrogel is currently drawing increasing attention [39,40].

Asli et al. studied the in vitro antibacterial activities of various molecular weight (0.4–0.6, 1.3, 2.6 and 4.0 kDa) chitosan against *S. aureus* isolated from bovine mastitis. The results indicated that the intramammary administration of the 2.6 kDa chitosan, both alone or in combination with tilmicosin, could reduce the bacterial colonization in a murine intramammary infections model. Besides, they found that the antibacterial activity of chitosan linked with the molecular weight and degree of deacetylation [28]. Braber et al. reported that the chitosan with a lower degree of deacetylation showed higher water solubility but lower antibacterial activity against *S. aureus* [41]. In our research, we should have selected chitosan with a higher degree of deacetylation to get more efficient antibacterial and treatment effect. However, chitosan with a high degree of deacetylation only dissolves in strong acidic surroundings. If the pH of the intramammary preparations was too low, they might lead to a strong stimulation of the mamma of cows. Therefore, the CMCS with high solubility in a different pH was chosen in our work. In the research of using natural polymers to promote wound healing, SA showed great potential due to its non-toxic, hydrophilic and biodegradable properties [42]. It was reported that the SA-povidone iodine material showed accelerated wound healing properties in a mouse model [42]. Raguvaran et al. indicated that SA-gum acacia hydrogels showed a healing effect and significantly reduced the toxicity of zinc oxide nanoparticles [43]. The clinical *S. aureus* mastitis is followed by inflammation and wounds, due to the wound healing promotion properties of SA, higher efficacy of tilmicosin SA nanogel could be expected.

### 3.2. Physicochemical Characteristics of TIL-SLNs and TIL-Nanogel

**Figure 2:**
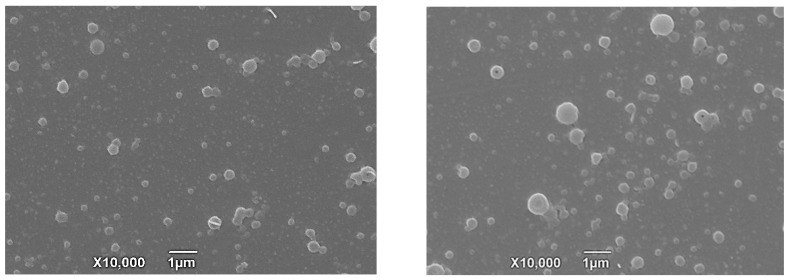
The scanning electron microscopy photographs of TIL-SLNs (left) and nanogel (right).

The SEM photographs show that the shape of TIL-SLNs and TIL-nanogel was a spherical shape with a relatively uniform distribution (Figure 2). The mean size, zeta potential and PDI of the TIL-SLNs were 311.33 ± 5.53 nm, −12.3 ± 0.05 mv and 0.306 ± 0.017, respectively, and the mean size, zeta potential and PDI of the TIL-nanogel were 481.57 ± 12.87 nm, 8.3 ± 0.06 mv and 0.424 ± 0.032, respectively. The LC and EE of the TIL-SLNs and TIL-nanogel were 20.58 ± 0.36%, 60.74 ± 2.55% and 23.33 ± 0.77%, 67.89 ± 3.01%, respectively. Comparatively, due to the encapsulation of SA, the LC and EE of the TIL-nanogel were a little higher than in the TIL-SLNs. Perhaps, because of the presence of cation substances (Ca^2+^, CMCS), the TIL-nanogel showed a weak positive charge. Whether the more advanced in vivo treatment efficacy of the nanogel than the TIL-SLNs was relative to the positive charge of the nanogel is worth studying. In addition, the SEM imaging of the TIL-nanogel showed a smoother and rounder appearance than the TIL-SLNs, which suggested that the SA and CMCS were coated on the surface of the TIL-SLNs.

**Figure 3:**
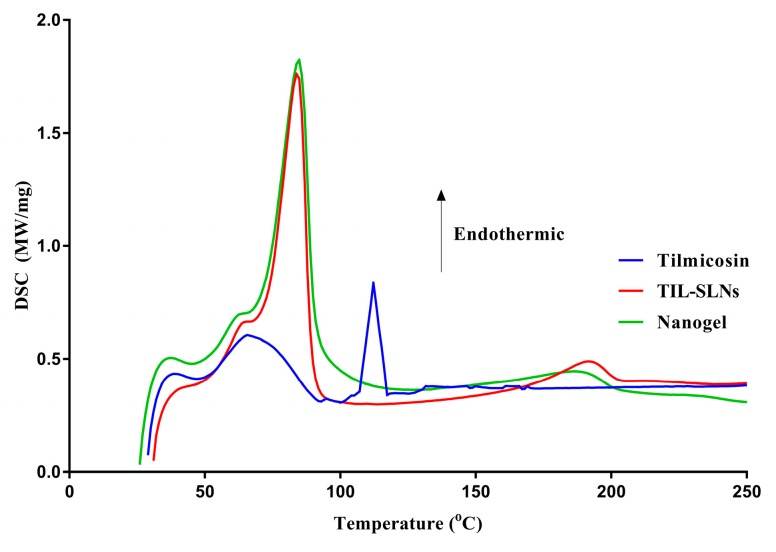
The differential scanning calorimeter (DSC) thermograms of pure tilmicosin, TIL-SLNs and TIL-nanogel.

The DSC curve (Figure 3) of tilmicosin showed a sharp peak at 112.2 °C indicating the melting point of pure tilmicosin was 112.2 °C (FAO). The TIL-SLNs and TIL-nanogel had sharp peaks at 83.8 and 84.8 °C, respectively, indicating that the melting point of carnauba wax was 83.8–84.8 °C, which agreed with the product description. However, no tilmicosin peak was observed in the TIL-SLNs and TIL-nanogel thermogram, indicating the disordered-crystalline phase of tilmicosin [44].

### 3.3. Sustained-Release Performance In Vitro

**Figure 4:**
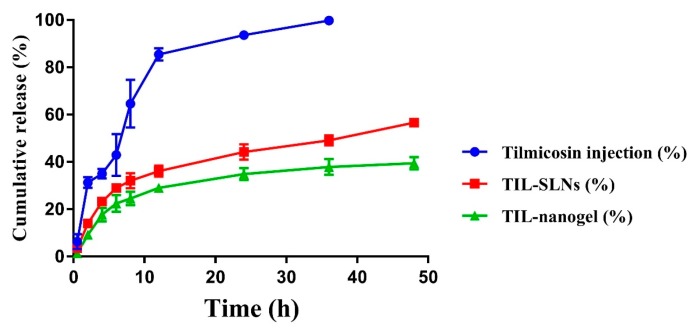
The cumulative release of TIL-SLNs and TIL-nanogel at pH = 7.5.

In vitro release performance of tilmicosin from the nanoparticles and nanogel were illustrated in Figure 4. The tilmicosin injection released 99.79% within 36 h. Compared with the injection, the TIL-SLNs and TIL-nanogel released 49.16% and 37.89% within 36 h, respectively, suggesting that both the TIL-SLNs and TIL-nanogel held prominent sustained-release performances. It also could be found that the TIL-nanogel showed slower release than the TIL-SLNs. Within 48 h, 56.63% of tilmicosin was released from the TIL-SLNs, while the cumulative release of tilmicosin from the TIL-nanogel was 39.43%.

Compared with the tilmicosin injection and TIL-SLNs, the TIL-nanogel showed the slowest release rate. The release mechanisms of tilmicosin from the TIL-SLNs might combine diffusion and erosion [45,46]. The burst release might be attributed to the swift dissolution of free and non-entrapped tilmicosin in the TIL-SLNs. For the TIL-nanogel, the release mechanism of hydrogels was illustrated clearly in previous research [47,48]. Firstly, water entered hydrogel matrix, the hydrogel swelled and the TIL-loaded carnauba wax SLNs started to be corroded. Then tilmicosin was diffused from the TIL-SLNs and crossed the swelling hydrogel. The release rate of the TIL-nanogel depended on the swelling rate of the hydrogel matrix and the erosion rate of the carnauba wax [45,47]. Due to the swelling limitation of the hydrogel matrix, the TIL-nanogel showed a slower release rate than the TIL-SLNs.

### 3.4. Stability

The preliminary influence factors test indicated that the TIL-SLNs were degraded by about 20% on the fifth day under high-temperature 40 °C, high humidity, high light or away from light (Table 2). These results indicated that the degradation was from the formulation factors of the TIL-SLNs rather than environmental factors. When the pH of the TIL-SLNs was adjusted from pH 9.1 to 6.0 by adding citric acid, the TIL-SLNs obtained good stability (Table 2). The influence factors test of the TIL-nanogel showed that the appearance, labeled quantity, size and LC of the TIL-nanogel did not change obviously under high temperature, high humidity and high light (Table 3). It suggested that storage stability of TIL-nanogel could be expected. 

The primary stability study proved that the TIL-SLNs was unstable at pH = 9.1. The in situ gelling preparation method of SA hydrogel with GDL (H^+^ source) not only could make uniform hydrogel, but also could adjust the pH to weak acidity. The influence factors study of the TIL-nanogel showed that the TIL-nanogel was stable under high temperature, high humidity and high light.

### 3.5. Antibacterial Activity Test In Vitro

**Figure 5:**
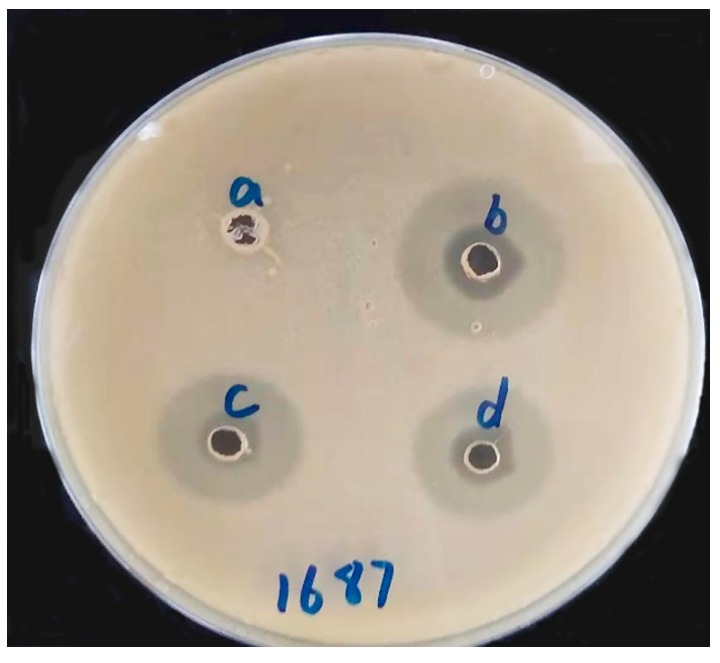
The inhibition zones of TIL-SLNs and TIL-nanogel against *S. aureus* 1687. Note: (a) control; (b) tilmicosin standard; (c) TIL-SLNs; (d) TIL-nanogel.

The antibacterial activity of TIL-SLNs and TIL-nanogel are presented in Figure 5. From the size of the inhibition zone, we could obtain that tilmicosin has antibacterial activity against clinical *S. aureus*. The inhibition zone of the control, tilmicosin standard, TIL-SLNs and TIL-nanogel were 0.0 cm, 2.17 ± 0.05 cm, 1.90 ± 0.08 cm and 2.07 ± 0.05 cm, respectively.

The purposes of the in vitro antibacterial test were to verify whether the TIL-SLNs and TIL-nanogel could improve the in vitro antibacterial activity of tilmicosin and if the preparation methods would influence the antibacterial activity of tilmicosin. The antibacterial test demonstrated that the TIL-SLNs and TIL-nanogel did not improve the antibacterial activity of tilmicosin in vitro (Figure 5). This might be because the diffusion of the TIL-SLNs nanosuspension and TIL-nanogel in agar was more difficult than the tilmicosin solution. Despite the fact that the in vitro antibacterial activity of tilmicosin was not improved, the inhibition zone of the various groups was similar. This indicated that the structure of tilmicosin was not changed in the processes of the preparation of the TIL-SLNs and TIL-nanogel.

### 3.6. Clinical Treatment Effects 

**Figure 6:**
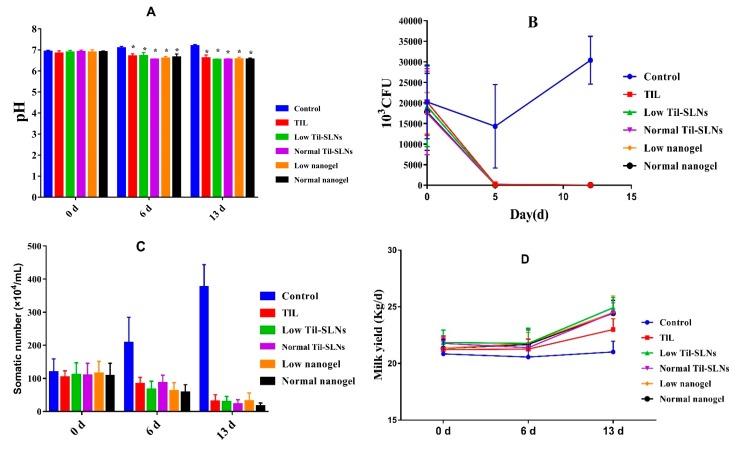
The change of pH, *S. aureus* counts, somatic number and milk yield, before and after breast perfusion. Note: (**A**) The pH of milk samples, * statistically significant from control (*p* < 0.05) by one-way analysis of variance; (**B**) *S. aureus* counts; (**C**) The change of somatic number; (**D**) The change of milk yield. Control: without treatment. TIL: treatment with commercial tilmicosin injection (300 mg/gland per day); Low TIL-SLNs: low dosage group of TIL-SLNs (150 mg/gland per day); Normal TIL-SLNs: normal dosage group of TIL-SLNs (300 mg/gland per day); Low nanogel: low dosage group of nanogel (150 mg/gland per day); Normal nanogel: normal dosage group of nanogel (300 mg/gland per day).

**Figure 7:**
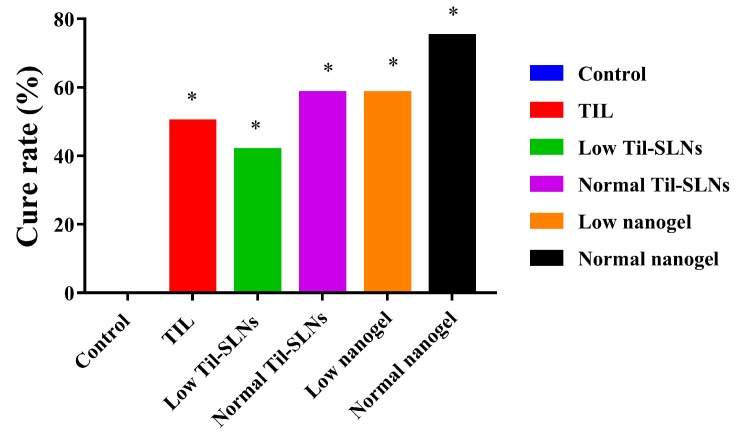
The cure rate of various groups after administration. Note: TIL: treatment with commercial tilmicosin injection (300 mg/gland per day); Low TIL-SLNs: low dosage group of TIL-SLNs (150 mg/mammary per day); Normal TIL-SLNs: normal dosage group of TIL-SLNs (300 mg/mammary per day); Low nanogel: low dosage group of nanogel (150 mg/mammary per day); Normal nanogel: normal dosage group of nanogel (300 mg/mammary per day). * statistically significant from control (*p* < 0.05) by chi-square test.

The milk samples of 72 cows with *S. aureus* mastitis were collected the day before administration, the 6th day and the 13th day after administration to evaluate the treatment effect of TIL-SLNs and TIL-nanogel. The pH changes of the milk samples before and after administration for five continuous days is shown in Figure 6A. The pH was in the range of 6.9–7.1 in control group, while the treatment groups of different formulations at the dosages of 150 or 300 mg tilmicosin/mammary were reduced from 7.1 to 6.5. The *S. aureus* counts in the control group showed the trend of rising and then falling. Compared with the control group, the *S. aureus* counts of treatment groups were reduced rapidly and were below the detection limit (Figure 6B). The SCC in the control group increased from 118.5 × 10^4^ cells/mL to 378.9 × 10^4^ cell/mL, and that of the treatment groups decreased quickly. Additionally, the mean SCC of the 300 mg/mammary per day nanogel group was less than other groups (Figure 6C). The results demonstrated that combination of TIL-SLNs with hydrogel (SA, CMCS) could improve the effect of tilmicosin against *S. aureus* mastitis. After treatment, the milk yield of cows also increased compared to that of cows in the control group (Figure 6D). It is worth mentioning that the reduction degree of pH, *S. aureus* counts and SCC of the TIL-SLNs group and nanogel group at the dosage of 150 mg/mammary per day were similar to those of the tilmicosin injection group at the dosage of 300 mg/mammary per day. The cure rate of the control group, tilmicosin injection group, low dose of TIL-SLNs group, normal dose of TIL-SLNs group, low dose of nanogel group and normal dose of nanogel group was 0.0%, 50.0%, 41.7%, 58.3%, 58.3% and 75.0%, respectively (Table 4, Figure 7). These suggested that the half-dose of TIL-nanogel (150 mg/mammary per day) could reach the same treatment effect as a normal dose of commercial tilmicosin injection and TIL-SLNs (300 mg/mammary per day).

The in vivo treatment experiment of mastitis demonstrated that tilmicosin was effective for clinical *S. aureus* mastitis since the effective rate was above 60% after treatment. It is worth mentioning that the TIL-SLNs groups and nanogel groups had a higher effective rate than tilmicosin injection. This suggested that the nanoparticle preparations could improve the treatment effect of tilmicosin against cow mastitis. Our result agreed with the previous research. Tilmicosin HSO-SLNs obviously improved the treatment efficacy of tilmicosin in a mouse model of mastitis as determined by lowering the colony-forming unit counts [49]. One of the reasons why the TIL-SLNs and nanogel had higher in vivo antibacterial activity than the commercial tilmicosin injection might be because the liquid environment of a cow mammary was beneficial to the uniform distribution of TIL-SLNs and TIL-nanogel. The most important aspect was that the cure rate of the low nanogel group (150 mg/mammary per day) was slightly higher than the tilmicosin injection group (300 mg/mammary per day). This is significant for reducing the dosage of tilmicosin and treatment costs if, as what our experiments proved, the combination of antibiotics with hydrogel (e.g., chitosan, SA) could improve the treatment effect and reduce the usage of antibiotics against mastitis. According to reports, the chitosan nanoparticles enhanced the uptake and transport capacity of the intestine [19] and improved the uptake values of polyoxometalates in breast tumoral cells [50]. If the prepared TIL-nanogel could improve the tilmicosin uptake by mammary epithelial cells is worth further study.

Meanwhile, the highest cure rate in this research was 75.0% after treatment for 5 days. The reason why *S. aureus* mastitis could not be cured completely, besides drug resistance, was perhaps the presence of *S. aureus* small colony variants (SCVs). Because of the lower metabolism level and growth rate of SCVs, many antimicrobial agents are ineffective against them [51,52]. The SCVs could change to a normal phenotype in their normal life cycle, thus leading to difficulty curing some infected cows [12]. According to reports, because of the ability to increase cell metabolism level, deferiprone and protoporphyrin could enhance the activity of antibiotics against SCVs of *S. aureus.* [53]. Combining nanogel with cell metabolism active agents might be a potential measure to remove the SCVs. Additionally, probiotic bacteria are thought to reduce intestinal colonization by pathogens and reduce susceptibility to infections. It was reported that the fengycin produced by *Bacillus subtilis* could interfere the Agr (accessory gene regulator) signal pathway of *S. aureus*, thus reduce the colonization of *S. aureus* in human and rat intestines [54]. We wonder if the bacterial against bacterial method (mammary perfusion *Bacillus subtilis*) could reduce the colonization of *S. aureus* in bovine mammary. In further research, the treatment effect of mammary perfusion fengycin or combination fengycin with nanoparticles is worth studying. Finally, the toxicity of nanoparticles always is a hot topic. Hart et al. indicated that carnauba wax nanoparticles could activate antigen-presenting cells without the production of potentially harmful inflammatory mediators [55]. Madureira et al. proved that the 0.15 mg/mL rosmarinic acid loaded carnauba wax nanoparticles did not affect DNA and the carnauba wax nanoparticles did not affect the growth of rats at dosage 10 mg/kg b.w. (body weight) after feeding for 14 days [56]. Although there was no shortage of research which proved the high safety of sodium alginate and chitosan nanogel [43,57], the safety of the tilmicosin hydrogel delivery system is still not reported. In view of this, the safety of TIL-SLNs and TIL-nanogel can be expected and needs further study.

## 4. Conclusions

The TIL-nanogel was developed by combining SLN technology with in situ hydrogel technology. The LC, sustained release and stability of the TIL-nanogel was superior to that of the TIL-SLNs. The TIL-nanogel at the dose of 150 mg/mammary per day showed a slightly higher cure rate than the commercial tilmicosin injection at the dose of 300 mg/mammary per day and an equal cure rate to the normal TIL-SLNs group at the dose of 300 mg/mammary per day. This study suggested that TIL-nanogel could reduce the dosage of tilmicosin for cow *Staphylococcus aureus* mastitis. The combination of hydrogel technology with the SLN technology will be a potential method for treating *Staphylococcus aureus* cow mastitis.

## Figures and Tables

**Table 1:** The dosage regimen of *S. aureus* mastitis treatment experiment.

Groups	Administration Volume (/Mammary Per Day)
Control Tilmicosin injection Low dose of TIL-SLNs Normal dose of TIL-SLNs Low dose of TIL-nanogel Normal dose of TIL-nanogel	10 mL physiological saline 1 mL tilmicosin injection (300 mg tilmicosin) + 9 mL physiological saline 3 mL TIL-SLNs (150 mg tilmicosin) + 7 mL physiological saline 6 mL TIL-SLNs (300 mg tilmicosin) + 4 mL physiological saline 3 mL TIL-nanogel (150 mg tilmicosin) + 7 mL physiological saline 6 mL TIL-nanogel (300 mg tilmicosin) + 4 mL physiological saline

**Table 2:** The influence factors test of TIL-SLNs (Mean ± SD, *n* = 3).

Sample	Influence Factors	5 d Labelled Quantity(%, pH 9.1)	5 d Labelled Quantity (%, pH 6.0)
TIL-SLNs	High temperature 40 °C High humidity High light Avoid light	80.8 ± 2.10 80.8 ± 1.98 80.3 ± 1.87 80.7 ± 2.13	100.7 ± 1.88 104.1 ± 1.54 103.0 ± 1.39 /

**Table 3:** The influence factors test of TIL-nanogel (Mean ± SD, *n* = 3).

Influence Factors		Time (d)		
		0 d	5 d	10 d
High temperature	Appearance	Milk white	Milk white	Milk white
	Labelled (%)	100.0	100.7	99.9
	Size (nm)	412 ± 6.5	436 ± 6.0	446 ± 4.5
	LC	23.5%	22.8%	21.5%
High humidity	Appearance	Milk white	Milk white	Milk white
	Labelled (%)	100.0	104.1	99.22
	Size (nm)	405 ± 5.2	412 ± 6.1	419 ± 4.6
	LC	23.1%	22.7%	22.8%
High ligh	Appearance	Milk white	Milk white	Milk white
	Labelled (%)	100.0	103.0	97.9
	Size (nm)	414 ± 4.3	438 ± 5.8	426 ± 6.1
	LC	22.9%	23.0%	22.5%

**Table 4:** The cure rate of *S. aureus* mastitis treatment experiment (*n* = 12).

Groups	Cure Rate (%)	Effective Rate (%)	Invalid Rate (%)
Control	0/12 (0.0)	0/12 (0.0)	12/12 (100.0)
Tilmicosin Injection	6/12 (50.0) *	8/12 (66.7)	4/12 (33.3)
Low Til-SLNs	5/12 (41.7) *	10/12 (83.3)	2/12 (16.7)
Normal Til-SLNs	7/12 (58.3) *	12/12 (100.0)	0/12 (0.0)
Low Nanogel	7/12 (58.3) *	10/12 (83.3)	2/12 (16.7)
Normal Nanogel	9/12 (75.0) *	12/12 (100.0)	0/12 (0.0)

* Statistically significant from control group (*p* < 0.05) by chi-square test.

## Supplementary Materials

The following are available online at https://www.mdpi.com/1999-4923/11/10/524/s1, Table S1: Factors and levels of the L9 (3^4^) orthogonal design, Table S2: The optimization of emulsifier by orthogonal experiment (L_9_3^4^).
