# Supplementary material for: Enhanced Treatment Effects of Tilmicosin Against Staphylococcus aureus Cow Mastitis by Self-Assembly Sodium Alginate-Chitosan Nanogel
Source: Pharmaceutics. 2019 Oct 12;11(10):524. doi: 10.3390/pharmaceutics11100524 (PMC6836059; doi:10.3390/pharmaceutics11100524)
Supplement: Supplementary file 1 [file pharmaceutics-11-00524-s001.pdf]

pharmaceutics

Supplementary Materials: Enhanced Treatment Effects of Tilmicosin Against *Staphylococcus aureus* Cow Mastitis by Self-Assembly Sodium Alginate-Chitosan Nanogel

Kaixiang Zhou, Xiaofang Wang, Dongmei Chen, Yuanyuan, Yuan, Shuge Wang, Chao Li, Yuanyuan Yan, Qianying Liu, Liwei Shao, Lingli Huang, Zonghui Yuan and Shuyu Xie

Table S1. Factors and levels of the L9 (3^4) orthogonal design.

Variable	Level		
	1	2	3
Type (A)	PVA	PVP	Poloxamer188
Concentration (B)	1%	2%	3%
Volume (C)	15 mL	20 mL	25 mL

Notes: PVA: Poly vinyl alcohol; PVP: Polyvinylpyrrolidone.

Table S2. The optimization of emulsifier by orthogonal experiment ($L_9 3^4$).

Sample	Type (A)	Concentration (B)	Volume (C)	LC (%)	Size	PDI
1	2	2	3	16.02 ± 0.85	1.339 ± 0.012	>0.5
2	3	1	3	16.52 ± 0.70	1.677 ± 0.009	>0.5
3	2	3	1	18.99 ± 1.97	5.847 ± 0.073	>0.5
4	1	3	3	20.90 ± 0.65	0.336 ± 0.005	0.368 ± 0.024
5	3	3	2	17.61 ± 0.95	5.347 ± 0.036	>0.5
6	3	2	1	17.04 ± 1.65	2.451 ± 0.013	>0.5
7	1	1	1	17.19 ± 0.98	0.481 ± 0.013	0.424 ± 0.032
8	1	2	2	15.47 ± 0.98	0.311 ± 0.005	0.306 ± 0.018
9	2	1	2	15.91 ± 1.23	1.419 ± 0.005	>0.5
k1	17.85	16.54	17.74			
k2	16.64	15.84	16.33			
k3	17.05	19.16	17.81			
R	1.21	3.32	1.48			
Optimum	A1	B3	C3			

Notes: k1, k2, and k3 are the average of grade for 3 levels in each factor; R is the different value between the max and mix of k1, k2, and k3 in each level. Abbreviations: LC: load capacity; PDI: poly dispersion index. Optimum formulation of TIL-SLNs: PVA, 3%, 25 mL.
